# Recruiting for research on sensitive topics in schools: an experience with Vaxcards, a collectable vaccine card game

**DOI:** 10.1186/s13063-021-05288-4

**Published:** 2021-05-03

**Authors:** D. S. Epstein, J. C. Enticott, H. J. Larson, C. Barton

**Affiliations:** 1 Department of General Practice, Monash University, Building 1, 1/270 Ferntree Gully Road, Notting hill, Melbourne, Victoria 3168 Australia; 2 Monash Partners, Advanced Health Research and Translation Centre, Melbourne, Australia; 3 The Vaccine Confidence Project, Department of Infectious Disease Epidemiology, London School of Hygiene and Tropical Medicine, London, UK; 4 Department of Health Metrics & Evaluation, University of Washington, Seattle, UK

**Keywords:** Vaccine hesitancy, Vaccine confidence, Vaccine consent, Vaccination, Vaccination rate, Vaccine schedule, Vaccine, Vaccine education, Preventative health, Health education, Health promotion

## Abstract

**Abstract:**

Undertaking recruitment for research in schools is an effective way to recruit young people for research participation but it is not without its challenges. Gaining access and coordinating many levels of different organisations and stakeholders whose cooperation and approval are crucial all add time and sometimes logistical challenges for the research team. In addition, recruiting around sensitive research topics can elicit additional barriers to successful research.

The research team aimed to conduct a pragmatic cluster randomised controlled trial involving schools in a local government region in Victoria, Australia, to assess the effect of a vaccination-based educational card game called “Vaxcards” on vaccine consent returns. Schools were contacted via phone and email to determine which staff member would best be a contact point for a face-to-face meeting to discuss the methods and purpose of the study. Email follow-ups were scheduled to follow up non-responsive schools and consent forms. The minimum required sample size was 13.

Of 31 eligible schools, 13 were recruited. The research team encountered several unanticipated challenges before achieving the recruitment target. The most common reasons for non-participation were being too busy with other commitments, concerns regarding the topic of vaccination being too sensitive, and concerns that key stakeholders in the school would not approve of the research topic of vaccination. One school required a review by a private research ethics board that rejected the study. Significant hesitancy and misinformation about vaccine science was observed that affected engagement with a small number of schools.

This paper highlights the challenges of recruiting schools in the context of public anxieties about vaccines and has several important learning lessons for successful recruitment about sensitive topics. This includes navigating approval processes for research in schools, the importance of local champions, dealing with misinformation and the importance of strong relationships and organisational trust.

**Trial registration:**

Australian New Zealand Clinical Trials Registry (ANZCTR) ACTRN12618001753246. Prospectively registered on 25 October 2018 8:24:21 AM

## Background

This paper describes the experiences of the research team recruiting young people from schools in Victoria for a trial of an intervention to improve return of consent to vaccinate forms. The research team focus especially on challenges in standardising recruitment strategies within schools and the impact on recruitment for research on a polarising topic such as vaccination.

There is increasing focus on recruiting participants from primary care and community settings in order to increase the applicability and translation of research findings [1–3]. Schools offer a trusted environment for participants that may make recruitment more successful [4] and are usually structured in an organised way so that access, attendance and recruitment processes can be achieved in a standardised way with the help of staff [4]. An intervention conducted in a school may provide students access to education, treatments or other health resources to which they would otherwise lack access [5].

Recruitment strategies should be reported in research for several reasons. This includes ensuring recruitment reports in evaluations are transparent and reproducible, documenting deviations in methodology (which can be common in pragmatic research designs), providing context and setting for complex interventions and ensuring accurate reporting of pragmatic data.

Further, there are several well-documented challenges to conducting research in the school setting. Gaining access to students for research in schools can be a barrier at many levels including different approval processes for research with government (public) schools, private and faith-based schools [5]. After gaining access, logistically coordinating many levels of authority, schedules and preferences of different schools and systems can be challenging [5–7]. The research team must also maintain and kindle a good relationship and work in collaboration with the stakeholders [5]. Students require parental consent to participate and researchers face additional challenges accessing and assuring this is achieved before commencing research [6].

This research was conducted in the School-based Immunisation Program in Victoria, Australia. In this program, schools are responsible for collecting consent forms that are provided by local government councils, who then provide health staff that visits the school later in the year to deliver the vaccines.

Several strategies and approaches to improve recruitment for research studies in school settings have been reported in the literature including developing key contacts, building relationships, logistical arrangements and facilitating trust in the research topic and team [4, 5, 8]. However, the research team encountered several additional challenges in the context of our research on what is currently a contentious and polarising issue, in vaccinations. Here the research team describe responses to these challenges and reflect on the implications of these actions in the context of further research in school settings in the context of vaccination research.

## Methods

### Description of the protocol

The study was a pragmatic, cluster randomised controlled trial involving 31 schools within a large local government area in the outer south east area of Melbourne, Australia. The trial was registered with the Australian Clinical Trials Register (ACTRN12618001753246) and granted ethics approval from Monash University Human Research Ethics Committee and the Department of Education, Victoria. The trial was instituted to test the effectiveness of a collectable card game to educate school-aged students about vaccine-preventable diseases and to incentivise students to engage in the vaccine schedule.

The principal investigator initially contacted schools that were randomised to the experimental group by phone to determine which staff member could be approached to act as a point of liaison for the school and to discuss the trial and provide information about the methods and purpose of the study. The research team tried to identify the coordinating staff member of the government vaccination program within each school, but found that this role varied by school and included staff whose primary roles included school nurse, year level coordinator, Vice Principal, Principal, student wellbeing officer or health subject teacher.

For our trial, this staff member would also be required to coordinate the logistical issues related to the delivery of the intervention at the school site. It is well known that local champions within organisations will provide much needed logistical help with recruitment [4]. It was important to identify the staff member responsible for coordinating the vaccination program as he/she will usually have an interest in vaccination and can champion the research within the school and provide a conduit to access higher level approval from school Principals or Councils as appropriate. Occasionally, this staff member was coordinating out of necessity and instead saw the research as an extra burden.

Heads of schools were provided an explanatory statement about the study and asked to provide signed consent for the school to participate. In Victoria, the heads of individual schools have the authority to provide permission for the research to be undertaken in their school. Work with our local champion, the research team tried to hold face-to-face meetings with the head of school to describe the study and answer any questions they may have about participating in it. Where a face-to-face meeting was not possible, we offered to provide this by phone or by email. The least ideal and least successful approach involved a receptionist or personal assistant passing on the information, in cases where the head of school was not accessible. An email was used to follow-up non-responsive schools and head of school consent forms.

After gaining consent for the school to participate in the research, individual parents in the experimental group were provided an explanatory statement about the study and the chance to opt-out if they did not want their child to receive the Vaxcards game pack. The trial information and opt-out forms were provided to parents/caregivers at the same time as the school provided information about the routine vaccination program and requested consent for the child to receive the standard vaccine schedule for that age/year level. It should be noted that the trial did not involve a change to the routine vaccine schedule for children this age and the researchers were not involved in the administration of the vaccination program.

Parents/caregivers could opt-out of receiving the incentive (a pack of Vaxcards), but still consent to their child receiving the vaccine schedule. On receipt of the study consent form, and with caregiver approval to receive the incentive, the school would provide one packet of Vaxcards to children at the time of vaccination.

Children attending schools in the control arm underwent their normal process of government school-based vaccination program including returning a government consent form; however, they were not offered the incentive. Following completion of the school vaccination program, schools in the control group were contacted by the research team to recruit them into the study with similar methods to the experimental group regarding participation in an identical web-based survey about vaccination hesitancy that takes an estimated 10 min, asking Likert-scale responses to 10 questions on beliefs about vaccine hesitancy.

Return rates of consent to vaccination forms were recorded by the local government council as per their standard protocols for this and formed the primary outcome variable for the trial.

The researchers then reflected on the recruitment and data collection processes and experiences post-study, the issues faced and the limitations and learnings from the study (Table 1).

**Table 1:** Characteristics of participating and non-participating schools

School	Characteristics
Participating	• Head of school or staff agrees to early face-to-face meeting out of interest in research/vaccination. • Local champion is interested in vaccination/student wellbeing • Head of school is interested in increasing vaccination participation
Non-participating	• Email contact only, no phone/face-to-face availability • Repeated disinterest or non-response from the key staff initially contacted. • Concern about the topic of vaccination being sensitive with school stakeholders • Multiple levels of approval required • Staff coordinating vaccination program in charge by necessity, not out of personal interest. • Head of school concern over parental backlash

## Results

### Outcomes of the recruitment process

Overall, recruitment was successfully completed within the very tight timeframes required given the “hard” deadline for the school vaccination program for this particular project, 2 months after the start of the year for new students. Of the 31 schools in the council, six schools did not participate in the council vaccination program, either because they did not have year 7 students or had special school vaccine schedules with varying ages in classes, and were excluded. One school had already completed the distribution of council consent forms and was excluded. Of the remaining 24 schools, *n*=13 (54.2%) consented to participate in the trial. Seven schools were randomised to the experimental arm and six to the control arm.

Survey responses were received from *n*=1221 individuals comprising *n*=248 parent/caregiver responses from 10 different schools and *n*=973 students from 11 different schools.

**Fig. 1:**
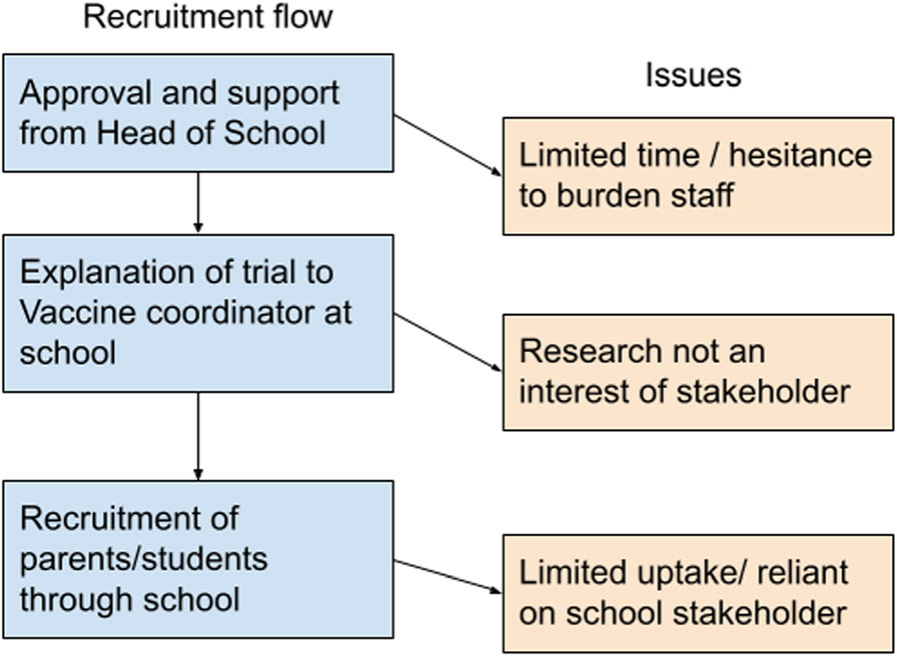
Simplified recruitment flow and common issues encountered

Trial results will be published in a separate manuscript. The simplified recruitment process and the places in the process where the issues were encountered are seen in Fig. 1.

### Reasons for non-participation

Of the 11 schools that did not participate when contacted, two schools failed to respond further after face-to-face or phone meetings and 9 declined participation. Schools were asked to provide comment on their reason for declining. The most common responses were that they were too busy with other commitments (*n*=4), concerned regarding the topic of vaccination being too sensitive (*n*=4), and concerned that key stakeholders in the school would not approve of the research topic of vaccination.

One school requested a further level of review from a faith-based school research ethics committee. This committee did not approve the study in this school setting and so the school was excluded. Reasons provided for this decision were that (1) “it was inappropriate for the school to send initial emails to parents and students inviting participation”, (2) “the survey contained questions which were deemed inappropriate”, and (3) “Given the process for communication and dissemination of information, the perception of the schools impartiality related to the topic of vaccination may be compromised”. It should be noted that the study was reviewed and approved by two separate, independent ethics committees prior to being reviewed by the faith-based ethics committee. This may reflect behaviour from the schools attempting to not further trigger or polarise vaccine-hesitant parents. Literature has shown pro-vaccine communication can, depending on the context, further polarise vaccine hesitancy [9]. It may be useful to consider ways to mitigate overt pro-vaccine interventions within high vaccine rejection communities to facilitate recruitment. It is also a reminder that schools have a role as gatekeeper to their students and have a strong influence on promoting or excluding research to students and families.

On a positive note, several school local champions were supportive of the project and engaged very well in integrating the trial and supporting the schools’ participation by approaching the head of schools with reassurance it would not be burdensome to their workflow and it would be beneficial for the school and students. The researchers found this support from local champions particularly helpful in the recruitment of schools.

### Protocol deviations

#### School request to exclude specific cards, in the Vaxcards pack

One school in the experimental group requested that the vaccination card representing the disease HPV be excluded from the pack. The head of school held concerns that the HPV vaccine could promote sexual promiscuity which was at odds with the school curriculum which promoted celibacy as a measure of preventing sexually transmitted diseases in preference to vaccination. The school suggested that the research team consider the evidence around links between vaccination of HPV and sexual promiscuity, which have been debunked [9]. It was decided by the research team to exclude the HPV card from the Vaxcards packs distributed within this school in order to retain the school in the study.

#### Other variations

To achieve fidelity, the research team provided a simple flow chart of actions to be used by staff to help guide when to distribute Vaxcards packs (upon return of consent form) and surveys (after vaccination day) as well as prompting to contact the research team with any questions via phone or email. One school failed to adhere to the protocol of the study and handed out packs to all students regardless of consent forms being returned or not. This reiterates the importance of pragmatic trials in order to identify real-world application problems with interventions. Given the pragmatic nature of the trial, data from this school was retained for analysis.

### Strategies adopted to increase school participation [10]

#### Strategy 1: Assuring confidence in the project

Reinforcing legitimacy, privacy and confidentiality to participants and organisations and ensuring fair and equal opportunity for all participants by actively offering participation are crucial. Careful consideration of wording and context of recruitment material and considering inherent perspectives of on-ground support staff is also important, especially if recruiting through organisations with scripted or pre-prepared recruitment packages.

#### Strategy 2: Finding a local champion

Identification and fostering of ground-level relationships early is key. Working with on-the-ground contacts can be important and helpful to improve research goals with less disruption to workflow, better communication and interest in the research.

#### Strategy 3: Utilising social proof

The research team identified the use of momentum during recruitment as advantageous, encouraging schools to join the growing list of other schools that are participating, by demonstrating the benefits to the school in joining their peer schools.

### Strategies to support protocol adherence

#### Strategy 1: Making the process easier

The research team had success implementing flow charts and stepwise instructions for ground-level staff on what is required, while also sending reminder emails or check-in calls around important milestones to make work requirements less burdensome for participants from organisations.

#### Strategy 2: Good communication and availability

The research team made it a priority to be available to answer phone/email concerns from anybody and prepare a running, regularly updated FAQ list to answer common requests or address problems encountered.

## Discussion

This paper aimed to describe the challenges in recruiting schools to a pragmatic randomised controlled trial on a sensitive topic within the Australian community, as it is in many communities internationally. Overall, recruitment was considered a success in the trial as the minimum estimated sample size was achieved and sufficient data was collected to analyse our primary and secondary outcomes. However, in achieving this, several barriers and challenges arose during the recruitment phase, which are discussed in detail below, and we reflect on our response to these challenges. Using the lessons learnt in this study, recommendations for designing, implementing and reporting recruitment within pragmatic trials, especially those conducted (i) in school settings and (ii) involving research about sensitive or polarising topics in the context of the CONSORT extension for pragmatic trials, were provided.

### Before recruitment started

To conduct research within the school environment, approval is required by many different stakeholders at different levels each with their own myriad of approval processes. Throughout this project, the research team had to submit our proposal to two additional ethics committees that delayed the start of the study. Schools could not be approached prior to achieving these approvals shortening the time available for recruiting school sites to the study. Strategies to mitigate logistical barriers within schools included leveraging existing ethics approvals and networks to help facilitate approval at the next level by providing social and ethical legitimacy.

One school-based ethics committee rejected our research proposal on the grounds of the topic being too sensitive for the school to uphold the school group’s impartiality to the topic of vaccination. This stance does not align with governmental and public health mandates of vaccination; however, it is important to remember stakeholders may choose to not participate and have the right to do so. Presenting sensitive research in an impartial way, allowing open communication and clearly outlining what is involved and the potential benefits to the wider community are key to recruiting as sensitively as possible. Nonetheless, there may be many reasons that some choose not to participate which need to be respected. It is important to design engagement strategies that address concerns in a non-confrontational manner.

### Recruiting about sensitive topics

The researchers did not anticipate the level of sensitivity to the issue of vaccination within government schools and the impact this would have on recruitment. First, an unanticipated sensitivity to discussions about vaccination was found within some schools that was very context-dependant, and had the potential to negatively impact on study recruitment, concordance with the trial protocol, and data collection from study participants [11]. When conducting research within schools, there may be staff concerns about discussing topics with participants and may raise ethical concerns that compromise studies that have received ethics approval [12]. Power imbalances between levels of approval may influence participation, informed consent or results within a community setting [11] (e.g. school and staff, school and student, student and parent and student). Within schools, researchers may additionally encounter specific school mandates on what should be taught [4]. There is also a real risk of self-selection bias and response bias amongst participants or organisations with strong personal opinions or beliefs.

### During recruitment

#### Finding local champions

It is important to have direct and open communication lines with these key stakeholders and make their job as easy and clear as possible, for example simple flow charts or step-by-step instructions. In schools, there is likely to be one member of staff that will be given the role of overseeing the research project. Attempting to find enthusiastic staff to champion your research may be the most important ingredient for a successful study. Staff who consider the project as an extra burden may require extra support in aligning the project to their usual business to increase engagement. It is worth noting here that relying on only one point of contact for a school may lead to a dead end on communication, bottleneck or hurdle for successful recruiting. Vaccination in general is a contentious issue that can illicit beliefs and responses that are not always in alignment between the research team and the participants. This highlights the importance of creating an environment for safe and non-judgemental discussion with stakeholders where they can express views on the subject and provide a chance for the researchers to engage with them in this. It is not so much a case of changing the stakeholder’s minds or beliefs, but creating an environment and relationship that allows the research to continue in an objective and impartial way.

#### Dealing with misinformation and disinformation

Unfortunately, addressing disinformation is a concern for researchers as well as the general public. During this project, the researchers were confronted by a head of school suggesting the research team consider evidence around links between vaccination of HPV and sexual promiscuity. Considering scenarios where you may encounter conflicting unscientific resistance will be crucial to successful research. Arguably, it is very important to have representation of all viewpoints in a study and the aim should be to help answer concerns about participation, not the content. This was actually a success with our project, as the researchers were able to answer concerns of one particular vaccine-hesitant stakeholder and get participation of a large population; however, this group ended being over-represented in our final sample, contributing a heavy weighting within the final cluster data. It can be difficult communicating with those with opposing agendas or opinions, but it is important to clearly lay out the purpose and methods of the study impartially and provide a safe place for direct questions and answers. However, it is likely to be counterproductive to argue or correct misinformation during recruitment. The role is to accurately present the purpose of the research, be flexible with requests and have representation. It is important to clearly script and prepare all research work that is to be passed onto participants, recognising that some channels may introduce bias.

### Lessons for real-world pragmatic designs

The CONSORT extension for pragmatic trials focuses on documenting and reporting on methods and settings that may be altered or designed for pragmatic variation. Within the recruitment section, there are no specific recommendations for pragmatic trials aside from documenting reasons for non-participation. When designing real-world pragmatic evaluations, such as this one, it is important to proactively consider pragmatic recruitment strategies in trial design to not only report non-participation, but to reduce non-participation with the strategies outlined.

Reporting on recruitment strategies is important for transparency, reproducibility and context. These recommendations include strategies to consider when recruiting within schools on sensitive or polarising topics such as vaccination.

### Strengths and limitations

The paper describes strategies we adopted before and during a trial to support recruitment of schools, school students and their parents as part of a study of a polarised issue in a school setting. The paper was written at the conclusion of the study, and we are mindful that it is often the most difficult experiences that stay with us. Also, being a pragmatic trial, several recruitment strategies had to vary from the set protocol to work around specific school structures, such as the request to remove the HPV card for one school, which is not ideal but considered better than no engagement with the trial at all. The reflections here draw on notes made during the study as part of research team meetings and reflections on interactions with stakeholders while in the field. Additional in-depth qualitative studies would be of value to explore reasons underlying the situations and challenges that were encountered and how the strategies in place or adopted were perceived by the participants and other stakeholders.

## Conclusion

Real-world research can present unanticipated challenges to a project that are not considered prior. It is very important for a team to consider possible setbacks and alternative strategies, create contingency plans and remain agile throughout the research process. It is also important to allow time to deal with unanticipated issues and keep communication with all stakeholders open and updated with changes.

## Data Availability

Available upon request
